# The exchangeability of shape

**DOI:** 10.1186/1756-0500-3-266

**Published:** 2010-10-22

**Authors:** Jean-Pierre AL Dujardin, Dramane Kaba, Amy B Henry

**Affiliations:** 1 Department of Medical Entomology, Faculty of Tropical Medicine, Mahidol University, Bangkok, Thailand; 2 Institute for Research and Development, Montpellier, France; 3 Institute Pierre Richet/INSP, BP. V 47, Abidjan, Ivory Cost; 4 Asia-Pacific Institute of Tropical Medicine and Infectious Diseases, University of Hawaii at Manoa, Honolulu, United States

## Abstract

**Background:**

Landmark based geometric morphometrics (GM) allows the quantitative comparison of organismal shapes. When applied to systematics, it is able to score shape changes which often are undetectable by traditional morphological studies and even by classical morphometric approaches. It has thus become a fast and low cost candidate to identify cryptic species. Due to inherent mathematical properties, shape variables derived from one set of coordinates cannot be compared with shape variables derived from another set. Raw coordinates which produce these shape variables could be used for data exchange, however they contain measurement error. The latter may represent a significant obstacle when the objective is to distinguish very similar species.

**Results:**

We show here that a single user derived dataset produces much less classification error than a multiple one. The question then becomes how to circumvent the lack of exchangeability of shape variables while preserving a single user dataset. A solution to this question could lead to the creation of a relatively fast and inexpensive systematic tool adapted for the recognition of cryptic species.

**Conclusions:**

To preserve both exchangeability of shape and a single user derived dataset, our suggestion is to create a free access bank of reference images from which one can produce raw coordinates and use them for comparison with external specimens. Thus, we propose an alternative geometric descriptive system that separates 2-D data gathering and analyzes.

## Findings

Morphometric techniques measure size, shape and the relation between size and shape (allometry). In practice, size and shape refer to a measurable part of the organism under study. A few anatomical landmarks (LM) available on a wing (or any measurable part of the body) do not completely describe the shape. However, provided there is operational homology [1] among individual LM, only a partial capture of shape is needed to allow valid comparisons among species

### Anatomical landmarks (LM)

Shape is described by new variables derived from raw coordinates of LM after Procrustes superimposition. These variables describing the shape of each specimen depend on the composition of the group under study. If other specimens (i.e. coordinates) are added to the analysis, shape variables must be recomputed accordingly [2,3].

### Size

To avoid the problem of multidimensionality, traditional systematists often select one single dimension to represent body size. For an insect, the length of the wing along its largest axis is frequently used as an estimator of body size [4-6]. Such relationship is often assumed rather than demonstrated [5].

#### Size variable: the centroid size

The centroid size (CS) is the square root of the sum of the squared distances from the centroid to each LM (see Gower, 1971 in [7]). It is a global size estimator informing about size changes in various directions. It is expressed in pixels, i.e. units relative to the resolution of the viewing device (most often a computer display). As a scalar it is less sensible to small digitization errors, and can be shared among systematists provided the pixels can be converted into absolute length units (inches, centimeters, millimeters, etc.).

### Shape

Not only in entomology, but also in many fields where morphometrics is applied, shape has been traditionally described as the ratio of one dimension to another. Although intuitively the ratio may appear capable of scaling for size, it often does not [8-11]. Moreover, the ratios introduce some well-known statistical drawbacks [9]. Angles also do not improve the situation since they are another kind of ratio [10].

#### Shape variables: the Procrustes residuals, the partial warps, the relative warps

In geometric morphometrics (GM), the shape of a configuration of LM is represented by their relative positions as contained in their coordinates after correction for size, position and orientation [7]. The statistical procedure is called Generalized Procrustes Analysis (GPA) [12]. Residual coordinates produced by GPA lie in a curved space [13,14], they must be further modified by a rigid rotation so that they can be studied using classical statistical techniques [15]. Resulting shape variables are called "partial warps" scores (PW). The PW, or their principal components, namely the "relative warps" (RW), may be used in classical statistical analyzes (a complete glossary of the many technical terms related to GM can be found at http://life.bio.sunysb.edu/morph). These transformations are computed relative to the consensus configuration derived from a specific group of samples, this thwarts mixing the final variables with other such variables computed from other individuals.

### Allometry

Geometric shape variables (PW) are not allometry-free variables (they are isometry-free variables). The tentative removal of the allometric effect on shape can be justified for intraspecific studies [8,16,17] and less so for interspecific comparisons, where allometric variation is likely to be part of the evolutionary differences relevant to systematics.

### Measurement error

Measurement error (ME) can be introduced at various steps of morphometric analysis [18]. The mounting technique of specimens or organs, the photographing conditions, and the user's skill to collect LM coordinates may produce artefactual variation. Generally, similar techniques are used to process similar organisms, and digital techniques of modern photography provide adequate resolution for correct recognition of LM under different conditions.

**Figure 1:**
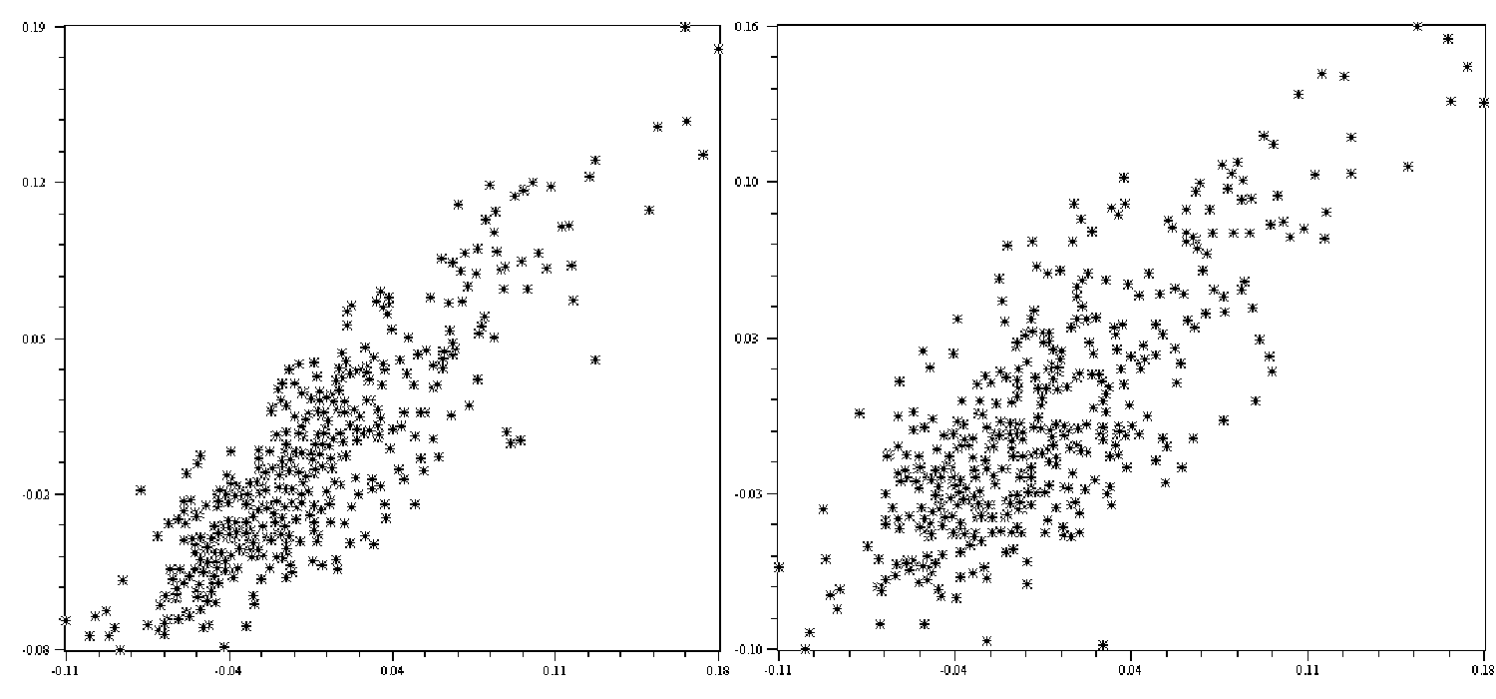
**Distances evaluated two times, by either the same user (left) or two different users (right)**. Dots (stars) are pair-wise Procrustes distances between female *Aedes aegypti *processed at 11 landmarks of the right wing. The distances were evaluated two times on the same set of specimens, by either the same user (left) or two different users (right). The repeatability of the measurements were estimated and shown Table 1. The user effect can be visualized going from left to right as a larger scattering of the distances around their expected values (identity). Procrustes distances were evaluated using the module COV of the CLIC software. The graph was obtained from the NTSYSpc-2.02™software.

**The "user effect" **When a single user repeats the measurements on the same specimens, the ME is generally not important. The "user effect" refers to the divergence between two users digitizing the same LM. Between two different users, the error is generally due to small but persistent differences in pointing to the exact location of some LM. We show the results of a repeatability [18] study on three different insect species (Table 1). The repeatability (R) could vary according to the user's skill and the quality of the anatomical LM [19], but it systematically decreased when two users were compared (Table 1). The effect was visibly amplified when looking at the final computation of Procrustes distances (Figure 1).

**Table 1:** Mean repeatability of landmark collection from the wings of three insect species

	One user	Two users
	**mean ± stdev**	**mean ± stdev**
		
*Glossina palpalis palpalis*	0.8053 ± 0.1101	0.6744 ±0.1623
*Glossina fuscipes fuscipes*	0.8099 ± 0.1315	0.6153 ± 0.2054
*Aedes aegypti *[43]	0.9206 ± 0.0303	0.8811 ±0.0632

Mean repeatability between the first and second measurements by the same user (first column "One user"), and mean repeatability between the measurements of a user versus the measurements of another user (second column "Two users"). The repeatability was measured as a Model II oneway ANOVA on repeated measures, where R is provided by the ratio of the between-individual variance and the total variance (module VAR of the CLIC package). stdev, standard deviation. The test was performed on the RW, and the average R over the first five RW are presented. The relatively high between-users repeatability in *Aedes aegypti *did not prevent a visible "user effect" on the Procrustes distances estimations (See Figure 1)

Reducing ME generally requires averaging repeated collections of the data [18]. However, such a laborious task might not be satisfactory when comparing very close specimens or groups, and ME may become a significant obstacle for different users [20,21].

### The taxonomic power of GM

The most important objection to the morphological concept of species is the existence of sibling (or isomorphic) species [22]. Sibling (or also cryptic) species are morphologically identical or nearly identical entities recognized as different species according to other, modern concept(s) of species. However, this objection to the typological concept (i.e. to "morphospecies") is weakened by the possibilities of modern quantitative shape comparisons [23-25]. Shape comparisons detect minimal morphological variations, which often are undetectable by traditional morphological studies and even by classical morphometric approaches. Cryptic species of insects showed distinct shapes in Triatominae [26-28], sandflies [29], parasitoid hymenoptera [23,30,31], fruit flies [32] and screwworm flies [33]. Morphometric discrimination is not confined to species determination, it has also been used to question species boundaries [34], or to synonymize controversial taxa [35].

### A geometric characterization system

Traditionally, morphometric traits have been introduced in dichotomous keys in the form of ratios, e.g. "the second antennal segment larger than the first one". GM does not use ratios, it is a powerful multi-characters approach able to derive quantitative information about morphological similarities. However, the consensus-dependent construction of shape variables prevents GM to be converted into a straightforward taxonomic tool [25,36,37].

Zelditch et al. [36] suggested identifying anatomical parts showing differences on D'Arcy Thompson visualization grids, then introducing ratios to taxonomic key. This proposition could be acceptable as long the GPA accurately identifies each LM displacement. However, the GPA considers the whole configuration and not individual LM. Moreover, extracting localized difference would mean some loss of information about shape variation, an unwanted effect when comparing conspecific populations or morphologically "indistinguishable" species.

Admittedly, the simplicity of classical taxonomic keys cannot be achieved with modern morphometric methods, and if one wants to use the full metric properties of the organisms, an analytical step cannot be avoided. Our suggestion for a geometric characterization tool is to separate the analytical step from the constitution of the data, in line with "partial disarticulation" of Bowker [38].

### Circumventing the "user effect"

A drastic solution to eliminate the user's source of ME is to eliminate the human user. The task of collecting LM is then automatized by dedicated software [39-41]. Nonetheless, it might be expected that various algorithms of image recognition could differ and show unequal performances. In the same way we describe a "user effect", a possible "software effect" could exist too. Since this effect (Table 1) is amplified in the final distances computation (Figure 1), and because the classification is based on distances, more errors are expected when data are derived from two users.

Our results (Table 2) show the assignation errors using either Procrustes or Mahalanobis distances. As expected, the error rate increased when coordinates were collected by two different users. In total, this "user effect" produced a two times increase in total error rate after Procrustes classification, and a more than ten times increase using Mahalanobis classification (Table 2).

**Table 2:** Assignation errors using landmark collection from the wings of two *Glossina *species

Species	Distances	One user	Two users
*Glossina p. palpalis*	Procrustes	2/44 (5%)	2/44 (5%)
	Mahalanobis	1/44 (2%)	8/44 (18%)
			
*Glossina f. fuscipes*	Procrustes	5/44 (11%)	10/44 (23%)
	Mahalanobis	1/44 (2%)	14/44 (32%)

Total errors	Procrustes	7/88 (8%)	12/88 (14%)
	Mahalanobis	2/88 (2%)	22/88 (25%)

Assignation errors according to the "one user" and "two users" procedures, and according to Procrustes and Mahalanobis distances. "One user" means that the same user digitized both the reference and the external specimens. "Two users" means that reference and external individuals were digitized by two different persons. Absolute values are the number of wrong species attributions out of the total of "unknown" specimens examined. For instance, "2/44" ( first row) means that 2 of the 44 external individuals known to be *G. p. palpalis *were wrongly assigned to *G. f. fuscipes*, according to the Procrustes distances.

The solution to the multiple users problem which is immediately applicable is limiting image digitization to a single user, either a human or a software (Table 3, steps 3 and 4), while still allowing images to be shared (Table 3, step 1).

**Table 3:** A "one user" procedure of metric identification

	Images (provided by multiple users)
Step 1	Obtaining reference images from a web data base
Step 2	Obtaining images of unknown specimens
	
	Digitization (performed by a single user)

Step 3	Digitizing the images of reference (reference coordinates)
Step 4	Digitizing the images of the unknown specimens (unknown coordinates)
	
	Classification

Procrustes	
Step 5	Pairwise Procrustes distances between each unknown and each reference image
	
Mahalanobis	
Step 6*	Computing shape variables on the combined sets of coordinates obtained from step 3 and one unknown specimen obtained from step 4
Step 7	Computing a discriminant model using the reference shape variables, exclusively (a partition of data from step 6)
Step 8	Entering to the discriminant model the shape variables of the unknown specimen (a partition of data from step 6)
Go to Step 6 for the next unknown specimen.	

General steps implemented in the CLIC package, relevant to the geometric approach: digitization (Steps 3 and 4, module COO of the CLIC package), the Procrustes classification (Step 5, module MOG of CLIC) and the discriminant analysis (Steps 6 and 7, module MOG) to identify organisms using mean reference pictures (Step 1) and own pictures (Step 2). The step 2 refers to the field and/or laboratory activities of the biologist: for entomologists, it generally requires the traditional tasks of collecting, dissecting and mounting insects. (*) To reduce multidimensionality, the number of shape variables (Step 6) can be reduced by selecting a set of few first relative warps (RW, i.e. principal components of partial warps). The MOG module automatically selects a number of RW lower than the smallest sample group.

### 2-D pictures database

Instead of coordinates which are affected by the measurement error, a reference database would contain the digital pictures from which coordinates can be collected. Then a single user having access to these reference pictures could include them with her/his own images and analyze the images together. This procedure eludes the production of coordinates by different users, though it does not address the errors due to different mounting and photographing techniques.

Thus, to identify morphologically close species and characterize populations, we suggest for GM a procedure separating data gathering from analyzes, i.e. a system consisting of a 2-D pictures database (Table 3, step 1), the extraction of relevant data (Table 3, step 3) and a related model of individual classification (Table 3, step 2 and steps 4 to 8; see next paragraph).

Conditions to provide useful images, such as a size scale (reticule), separation of sexes or the need for published references, are described at the CLIC web page http://www.mpl.ird.fr/morphometrics/clic/index.html. Since the CLIC bank is dedicated to cryptic species, only images which have been the material of a published work would be accepted. Furthermore, to take into account the environment, reference images should be labeled with not only the species but, ideally, the geographic origin, the date of capture, and other parameters defining their habitats.

### Classification

Where specific canalization of shape is efficient, we expect any specimen to be more similar to other specimens of the same species than to specimens belonging to different species [42,43]. The species classification as implemented in the CLIC package would then rely on the estimation of metric distances and related attribution algorithm. Classification techniques making use of artificial intelligence [44-46] are not considered here. When adding supplementary data to an analysis performed on reference data, the supplementary data are assigned to the reference group with which they have the shortest distance. The shortest distance might be however an important one and actually outside the mean distance among the members of that group. Thus, assignment to a given class, i.e. "discrimination" in a statistical sense [47], does not necessarily mean belonging to that class ("identification" in the biological sense).

#### Procrustes distances

The Procrustes distances are based on a minimum criterion (GPA is based on a least-squares algorithm). They are computed in a curved space, so that they are not Euclidean distances. An Euclidean distance is simply a line drawn between two points on a plane, and can be computed from the coordinates of these points by the well-known Pythagorean theorem [36].

The MOG module of the CLIC package allows the introduction of unknown specimens, and then performs a first classification named "Procrustes classification". It is based on pair-wise Procrustes distances of each unknown with the average image of each reference species, as well as with each reference image separately. The direct shape comparison between individual configurations could appear as a relevant technique for classification of unknown specimens. In our example, the total error rate ranged from 8% to 14% according to the "one user" or "two users" modes, respectively (Table 2). However, this classification does not take into account the dispersion ellipses of the reference groups. In our approach, we want to assign unknown individuals to reference groups. Their dispersion ellipses may differ for artefactual (sampling process) or biological reasons (different correlations among variables), and produce undue overlapping or similarities with other specimens.

#### Mahalanobis distances

This influence of intragroup variation is taken into account with Mahalanobis distances by standardizing the within group variance [47]. Mahalanobis distances may be presented as Euclidean distances computed using the discriminant factors derived from either PW or RW as input variables. In the CLIC procedure (Table 3), the discriminant model is computed between the reference images only (Table 3, step 7), the unknown specimens are then added as supplementary data one by one (Table 3, step 8), and the shape variables used in this classification technique are computed relative to the consensus including the single unknown specimen (Table 3, step 6). The "one by one" procedure is mandatory. Should a large number of unknown, external individuals be entered at once and shape computed from the grand total, the external individuals would modify the total consensus, which could reduce the discrimination between references and alter the classification power.

The Mahalanobis classification is a powerful technique, but very sensible to possible artifacts and/or outliers: it produced the best result in the "one user" procedure (2%, see Table 2), the worst one elsewhere (25%, see Table 2).

#### Software

The modules of the optional CLIC package http://www.mpl.ird.fr/morphometrics/clic/index.html have been shortly described previously [11,25]. Similar, complementary or additional analyzes can be performed using other freely available software, most of them listed in the main GM web page: http://life.bio.sunysb.edu/morph.

## Conclusion

Information systematists could expect from GM is determining whether populations are drawn from multiple species and how they can be discriminated [36]. Here we suggest the use of GM to classify unknown specimens according to known reference species, and we show that to reduce artefactual classification errors, both unknown and reference specimens should be digitized by a single user. This is possible if the reference material is made available to the user from a free access online bank of images. Instead of transporting specimens from one user to another, their images can be made available thanks to web based technologies. The solution proposed here is mostly applicable to 2-D data that can be collected from photographs.

We suggest that the pictures deposited in the bank of reference images be labeled with geographic locality and data of collection time, this will provide the possibility for investigating intraspecific variation. Population structure studies are then also possible.

## Availability and requirements

1. Project name: CLIC (Collection of Landmarks for Identification and Characterization)

2. Project home page: http://www.mpl.ird.fr/morphometrics/clic/index.html

3. Operating system(s): Uploading images is platform independent. The CLIC package is currently available for Windows and Linux platforms only.

4. Programming language: HTML, TclTk (CLIC package)

5. Other requirements: The uploading process is currently performed by the author of the CLIC initiative. Images can be sent using for instance an online file sharing software connected to the dujardinbe@gmail.com email address.

6. License: The CLIC package is under GPL license.

## Competing interests

The authors declare that they have no competing interests.

## Authors' contributions

JPD designed the HTML CLIC page at http://www.mpl.ird.fr/morphometrics/clic/index.html, and wrote the source code of the CLIC package modules. JPD and AH redacted the paper. DK made the repeatability study on *tsetse *flies, and AH on *Aedes *mosquitoes. The authors read and approved the final manuscript.
